# Fabric-Templated Co-Deposition of Nanofiber-Reinforced Composite Films with Ordered Surface Arrays for Ultrasensitive Wearable Sensing

**DOI:** 10.3390/polym18182278

**Published:** 2026-09-17

**Authors:** Yixiao Wu, Yuchen Luo, Ziyi Xiong, Sile He, Lin Yang, Chong Zhao, Kun Yan

**Affiliations:** 1 School of Chemistry and Environmental Engineering, Wuhan Polytechnic University, Wuhan 430023, China; 2 School of Environmental and Chemical Engineering, Zhaoqing University, Zhaoqing 526061, China; 3 Key Laboratory of Textile Fiber & Product, Ministry of Education, Wuhan Textile University, Wuhan 430200, China

**Keywords:** chitosan, PVA-*co*-PE nanofiber, nanocomposite, ordered array structure, wearable material

## Abstract

Fabricating polymeric films with ordered surface microarrays is of great significance for wearable sensing applications, yet it remains challenging to achieve scalable, cost-effective, and biocompatible architectures. Herein, we report a fabric-templated co-deposition strategy to construct nanofiber-reinforced composite films with well-ordered surface arrays. The approach utilizes woven cotton fabrics as soft templates, where capillary action directs the deposition of chitosan/poly (vinyl alcohol-co-ethylene) nanofiber mixtures within the microscale grooves, enabling direct replication of the array patterns after solvent evaporation and alkaline crosslinking. The resulting films exhibit hierarchical microstructures with uniform nanofiber distribution, strong hydrogen-bonding interactions, and tunable surface topographies. The optimized composite (Chit/NF_1.5_) achieves well-defined ordered surface arrays, enhanced mechanical flexibility, and stable structural integrity. After surface metallization, the array-patterned films demonstrate a piezoresistive sensing performance, with a 2.6-fold enhancement in current variation and a 3.4-fold higher sensitivity particularly in a low-stress region (0–0.15 N) compared to flat films. A dual-mechanism response involving contact-area enlargement at large strains and nanofiber-mediated conductive pathway formation at small strains is proposed to explain the ultrasensitivity. This work provides a versatile and scalable route for engineering ordered surface architectures, holding great promise for wearable health monitoring, smart electronics, and biomedical devices.

## 1. Introduction

Creating polymeric films with ordered surface array structures holds significant research value in advanced materials science [1,2], as the synergy between the constituent materials and the surface architectures enables unique functionalities such as enhanced sensitivity, selective wettability, and anisotropic conductivity for broad applications in sensing [3,4,5], healthcare [6,7], and environmental remediation [8,9]. For instance, for wearable sensing applications, the surface array architectures can endow the films with stress-sensing capabilities [10,11,12]. Moreover, the controllable contact area and well-defined repeating units of array patterns enable the quantification of external stimuli, thereby facilitating real-time sensing and ultrasensitive detection [13,14,15]. Recently, a variety of strategies have been developed to fabricate ordered surface arrays across length scales ranging from nanometers to micrometers [16], and machine learning technology is being employed to assist in improving detection accuracy [17,18]. These include molecular self-assembly, chemical deposition, lithographic etching, as well as external-field-assisted approaches (e.g., optical [19], electrical [20], or magnetic [21] field induction) for constructing more sophisticated and finely resolved structures [22,23,24]. However, most of these methods typically require the prefabrication of molds or masks, rendering the overall process relatively complex and costly [25,26]. Although three-dimensional (3D) printing offers a template-free alternative, its practical application is constrained by limited printing efficiency, which hinders large-scale production within a short period [27]. Therefore, the development of a rapid and efficient strategy for fabricating array-structured films is of great significance.

Conventional fabrics are typically fabricated by weaving or knitting bundles of oriented microfibers, which are assembled into hierarchical structures. Such fabrics can be naturally regarded as soft templates, providing a versatile platform for the fabrication of films with ordered surface array architectures [23]. For instance, Wang et al. utilized a silk fabric as a template to prepare PDMS-based array films that can serve as large-area sensors [28]. Similarly, N. Khalili et al. reported the fabrication of analogous 3D micropyramidal structures using micropatterns mold [29]. Although PDMS-based materials offer satisfactory processability, their non-breathable nature and poor biodegradability severely restrict their broad applications for biomedical use [30]. Therefore, creating biodegradable porous composite films holds significant research importance for applications in wearable and biomedical materials [31,32]. Chitosan hydrogel is widely recognized as a promising material for biomedical and flexible electronic applications, owing to its natural abundance, biocompatibility, film-forming properties, and excellent skin conformability [33]. Nevertheless, its inherent mechanical fragility poses a significant barrier to practical use. Incorporating nanofibers into chitosan matrices has emerged as an effective strategy to simultaneously reinforce mechanical strength and tailor porous structures [32,34]. However, controlled spatial distribution of nanofibers within the hydrogel network remains challenging because random dispersion or aggregation compromises interfacial interactions and leads to structural inhomogeneity and reduced performance [35,36]. Existing alignment techniques including magnetic-field-assisted and self-assembly approaches are often hindered by operational complexity and substrate-specific constraints [37]. These limitations call for a facile and scalable strategy to engineer tailored nanofiber distributions and thereby achieve surface-patterned films with high sensitivity and reliable signal transduction for wearable sensing platforms.

This study proposes a capillary-driven co-deposition method on woven fabrics to fabricate nanofiber-reinforced polymer films with ordered array microstructures. The bottom-up approach utilizes the inherent porosity of woven fabrics to guide the directional arrangement of a chitosan/PVA-*co*-PE nanofiber mixture during solvent evaporation, resulting in an interpenetrating network and ordered surface arrays. Conductive coatings and bioactive molecule functionalization further enhance membrane multifunctionality. This work integrates nanofiber distribution control with scalable hydrogel fabrication via a textile-templated solvent-evaporation platform, offering a versatile route to hierarchically structured composites that address reinforcement challenges and enable smart materials for personalized healthcare and wearable electronics.

## 2. Materials and Methods

### 2.1. Materials

Chitosan (deactylation of 85%, 200 kDa) and poly (vinyl alcohol-co-ethylene) (PVA-co-PE, 44% ethylene unit) masterbatches were received from Sigma-Aldrich (Milwaukee, WI, USA). The CAB with butyryl content of 35–39% was purchased from Acros Chemical, Pittsburg, PA, USA. The fluorescein isothiocyanate (FITC, average molecule weight of 4000) was purchased from Shanghai Maclin Biochemical Technology Co., Ltd. (Shanghai, China). All other chemicals (analytical grade) were used without additional treatments.

### 2.2. Preparation of Chitosan/PVA-Co-PE Nanofiber Mixtures

The fabrication of PVA-*co*-PE nanofiber suspension was prepared via a melt-phase separation method combined with suspension coating techniques established in prior research [38]. Initially, pelletized mixtures of PVA-*co*-PE and cellulose acetate butyrate (CAB) at 20:80 mass ratio were introduced into a Leistritz co-rotating twin-screw extruder (MIC 18/GL 30D, Leistritz AG, Nuremberg, Germany, 18 mm barrel diameter) operating at 12 g/min feed rate (190–230 °C) throughout the process. Subsequently, the molten blend underwent extrusion through a die with a 25:1 draw ratio, followed by immediate water quenching to ambient temperature. To isolate nanofibers, CAB matrix removal was achieved through 24 h Soxhlet extraction using acetone solvent. Post-drying, the purified nanofibers underwent 6 h dispersion in isopropanol/water medium using high-shear mixing to generate homogeneous suspensions. The chitosan solution (1%, *w*/*v*, pH~5) was obtained by dissolving chitosan flakes in dilute HCl to a final pH of approximately 5.5, following a previously reported procedure [39].

### 2.3. Preparation of Chitosan/PVA-Co-PE Nanofiber Nanocomposite Films

The chitosan/PVA-*co*-PE nanofiber mixtures were prepared by adding various amounts of PVA-*co*-PE nanofibers into chitosan solution and magnetically stirring for 4 h to obtain a homogeneous mixture with final nanofiber concentrations of 1%, 1.5%, and 2%. The mixtures were then sprayed on a typical polypropylene (PP) melt blown woven fabric template with the assistance of compressed air to obtain the final patterned nanofibrous films with the coverage densities of 1.0 g/m^2^. After the solvent evaporation and subsequently until constant weight (R.T., 12 h), the films were crosslinked in 1 M NaOH solution for 1 h. The resulted films were then washed with deionized water for several time (to neutral) and coded as Chit/NF_1_, Chit/NF_1.5_, and Chit/NF_2_, respectively. The bare chitosan (Chit) and PVA-co-PE nanofiber (NF) were prepared as the control.

### 2.4. Wearable Sensing Performance

Prior to the sensing characterization, all film samples were sputter-coated with a gold layer for 15 min under the following conditions: sputtering current of 0.5 mA and chamber pressure of 0.1 Pa. Two film strips were individually attached to conductive adhesive tapes and subsequently stacked together to assemble the pressure sensing device. The compressive cyclic tests were conducted over 25 successive loading–unloading cycles (0.5 mm/min; 0–2.5 N), and the corresponding current variations were continuously recorded throughout the testing process.

### 2.5. Characterizations

The film microstructures were examined by scanning electron microscopy (SEM; VEGA3 LMU, TESCAN, Brno, Czech Republic), and the macroscopic morphologies were recorded using a digital camera and an optical microscope (MVX10, Olympus, Tokyo, Japan). Attenuated total reflection Fourier-transform infrared spectroscopy (ATR-FTIR, Nicolet 6700, Thermo Scientific, Waltham, MA, USA) was employed to characterize the chemical structures and surface compositions across the spectral range of 400–4000 cm^−1^. Mechanical properties were evaluated using a universal testing machine (CMT6350, Shenzhen, China) with a tensile rate of 1 mm/min, and all specimens were prepared to standardized dimensions to ensure reproducibility and measurement accuracy.

## 3. Results and Discussion

### 3.1. Soft Fabric-Templated Co-Deposition of Chit/NF Nanocomposite with Ordered Surface Array Architectures

**Figure 1:**
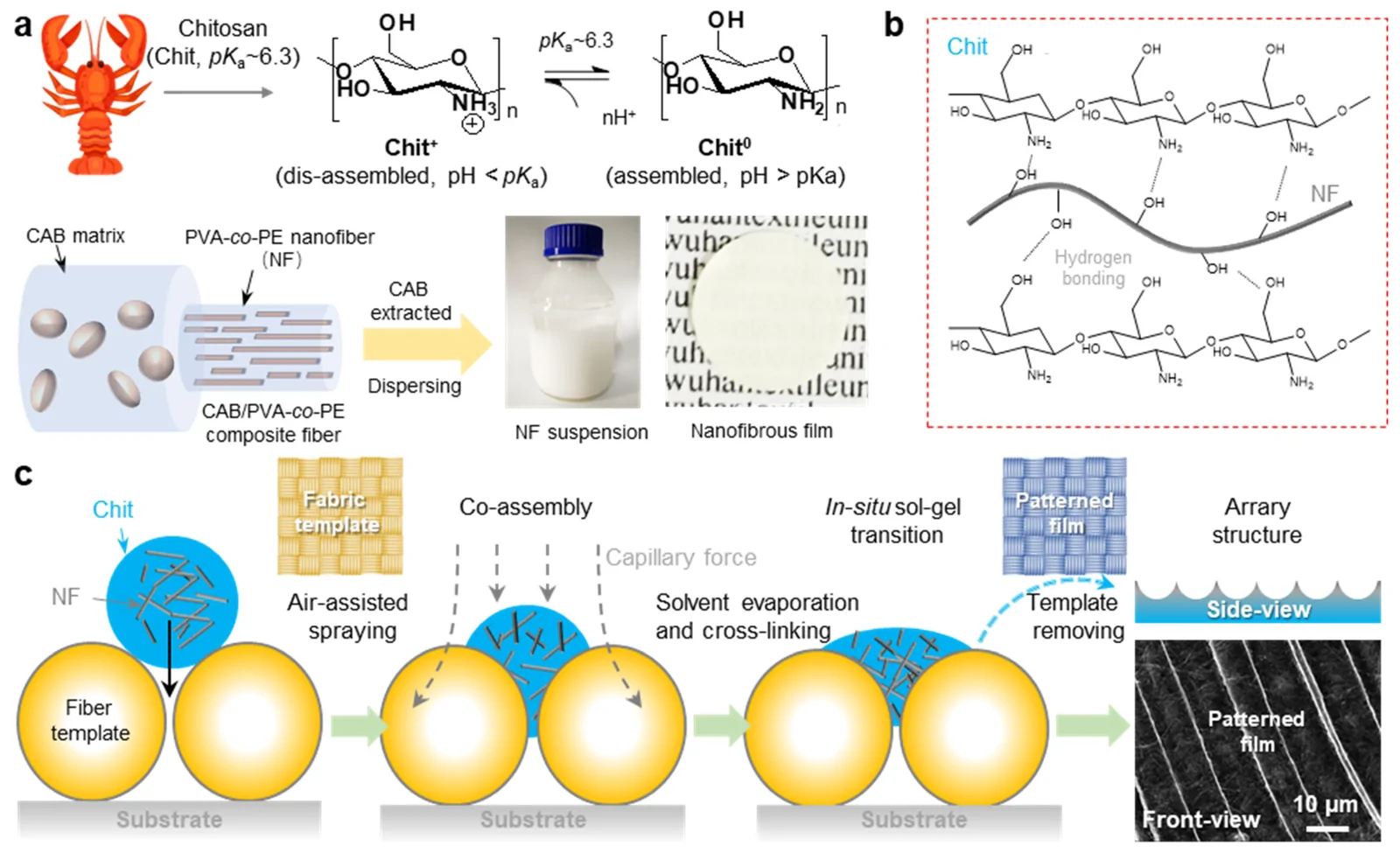
(**a**) Preparation and chemical structures of chitosan (Chit) and melt-extruded PVA-*co*-PE nanofibers (NF). (**b**) Molecular interactions between Chit and NF. (**c**) Schematic representation of the fabric-templated co-deposition strategy showing the formation of Chit/NF nanocomposite films featuring well-ordered array surface patterns.

The nanocomposite film was prepared using chitosan (Chit) and PVA-*co*-PE nanofibers (NF) as raw materials. As shown in Figure 1a, chitosan is a natural polymer extracted from shrimp shells that exhibits excellent physicochemical properties, and its amino groups can be protonated (pKa~6.3), conferring specific pH-dependent sol-gel transition responsiveness. In addition, the PVA-*co*-PE nanofibers were fabricated via a sea-island melt-spinning technique developed by our team [38]. After phase separation and acetone washing to remove the sea phase, the obtained nanofibers can be evenly redispersed into a uniform suspension, with an average diameter of approximately 412 ± 49 nm (SEM images of the nanofibers and their diameter distribution histogram are provided in Figure S1). Figure 1b illustrates the strong hydrogen bonding between chitosan (Chit) and nanofibers (NF) via hydroxyl and amino groups, which facilitates the formation of stable nanofiber-reinforced composite films. Figure 1c presents a schematic illustration of the surface architecture replication from the woven fabric template onto the nanocomposite film. The preparation process is primarily achieved by adjusting the concentration ratio of the mixed solution coupling with an airflow-assisted spray coating approach. The mixture is induced by capillary action between the template fibers to infiltrate the inter-fiber voids and then dried upon solvent evaporation. Subsequently, the composite film can be peeled off from the template surface and physically crosslinked with alkaline solution. The resulting structure is shown in the SEM image on the right side of Figure 1c, demonstrating that locally oriented alignment structures can be readily realized by this fabric-templating method [28]. This approach is simple and convenient, avoids the need for customized complex templates, and is more amenable to large-scale production, thereby providing a new strategy for regulating the surface microstructure of nanofiber-reinforced composite films.

**Figure 2:**
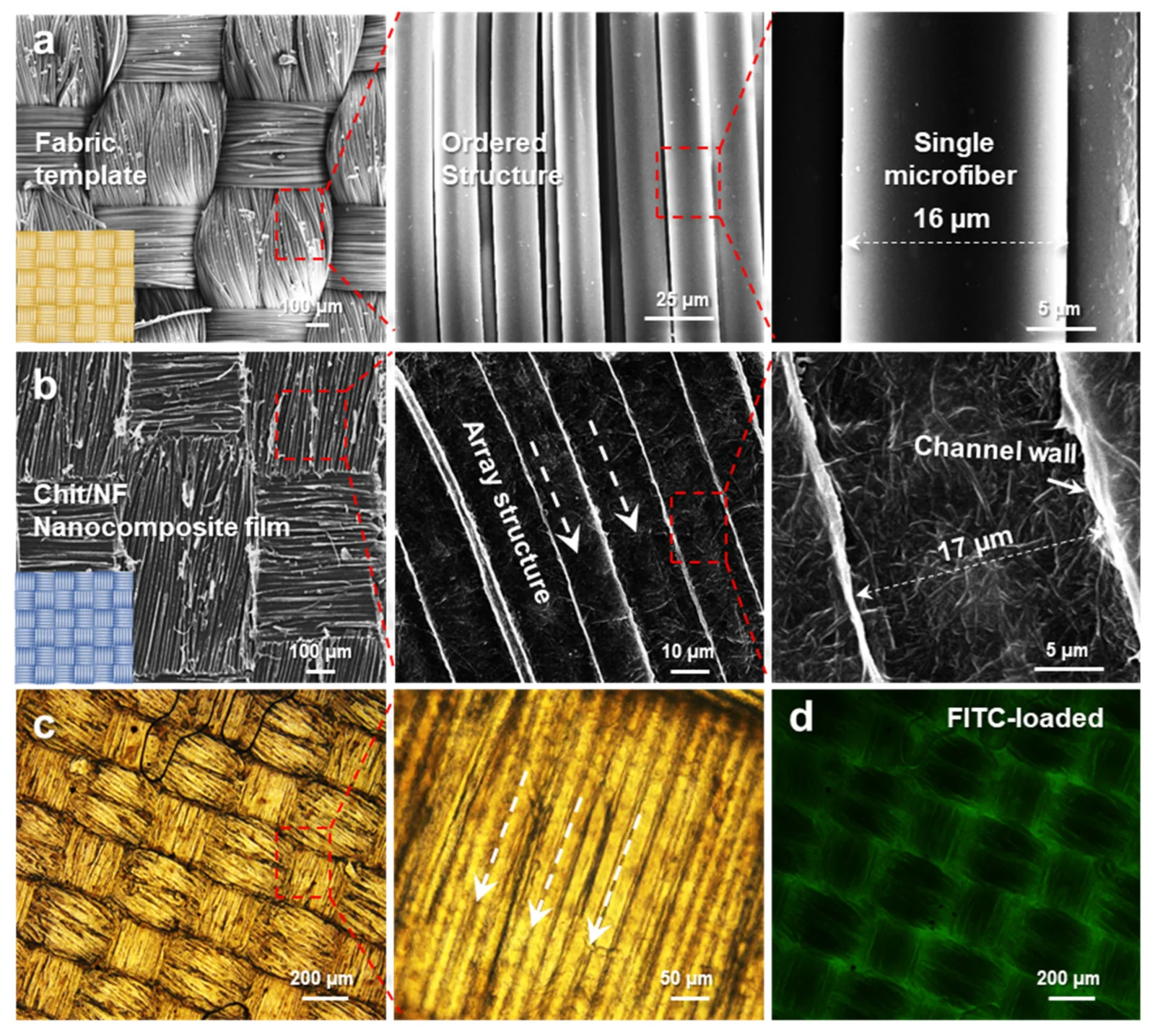
(**a**) SEM image of the pristine fabric template. (**b**) SEM images of the patterned Chit/NF films at different magnifications (insets showing the corresponding weave and array patterns). (**c**) Brightfield and (**d**) fluorescence microscopy images of the patterned films (arrow indicates the alignment direction); FITC staining confirms the successful loading of dye molecules within the arrayed microstructures.

The surface morphology of the natural fibric templates is presented in Figure 2a, wherein the woven or textured substrates feature well-defined microscale grooves that serve as topographical constraints to direct the guided co-deposition of the Chit/NF mixture. The soft fabric is conventionally woven from cotton yarns, each comprising numerous individual cotton fibers aligned in parallel. This inherently hierarchical structure provides an accessible, low-cost, and sustainable bio-template. As highlighted in the inset of Figure 2a, each cotton fiber exhibits a smooth, hydrophilic surface with a uniform diameter of approximately 16 µm, thereby functioning as a native aligned microstructure template that promotes the oriented deposition of the composite components. Intriguingly, these groove features are faithfully transcribed onto the Chit/NF nanocomposite film, yielding highly ordered surface array microstructures with excellent pattern fidelity (Figure 2b). The nanofibers are homogeneously distributed throughout both the channel walls and the bulk film matrix, forming an interpenetrating network. This unique architecture is anticipated to substantially reinforce the mechanical robustness of the composite films by facilitating uniform stress distribution and enhancing interfacial load transfer efficiency [40]. To gain deeper insights into the surface pattern formation, large-field optical images (Figure 2c) and corresponding fluorescence micrographs (Figure 2d) are provided. Both imaging modalities consistently reveal the presence of well-ordered surface arrays across a macroscopic scale, underscoring the high reproducibility of the templating process. More importantly, the fluorescence image of FITC-loaded Chit/NF confirms that diverse bioactive molecules (e.g., fluorescein isothiocyanate, FITC) can be effortlessly integrated into the film matrix via a co-deposition strategy, preserving their fluorescent functionality without detectable quenching. Overall, these findings establish that the fabric-templated co-deposition approach represents a versatile and scalable route under mild ambient conditions for engineering patterned composite films and demonstrates considerable broad potential applications ranging from high-performance wearable strain sensors that require durable and conductive surface textures [41] to advanced microneedle-based transdermal drug delivery systems that demand precise micro-architectural control [42].

### 3.2. Characterization and Structural Formation Mechanism of the Nanocomposite Films

**Figure 3:**
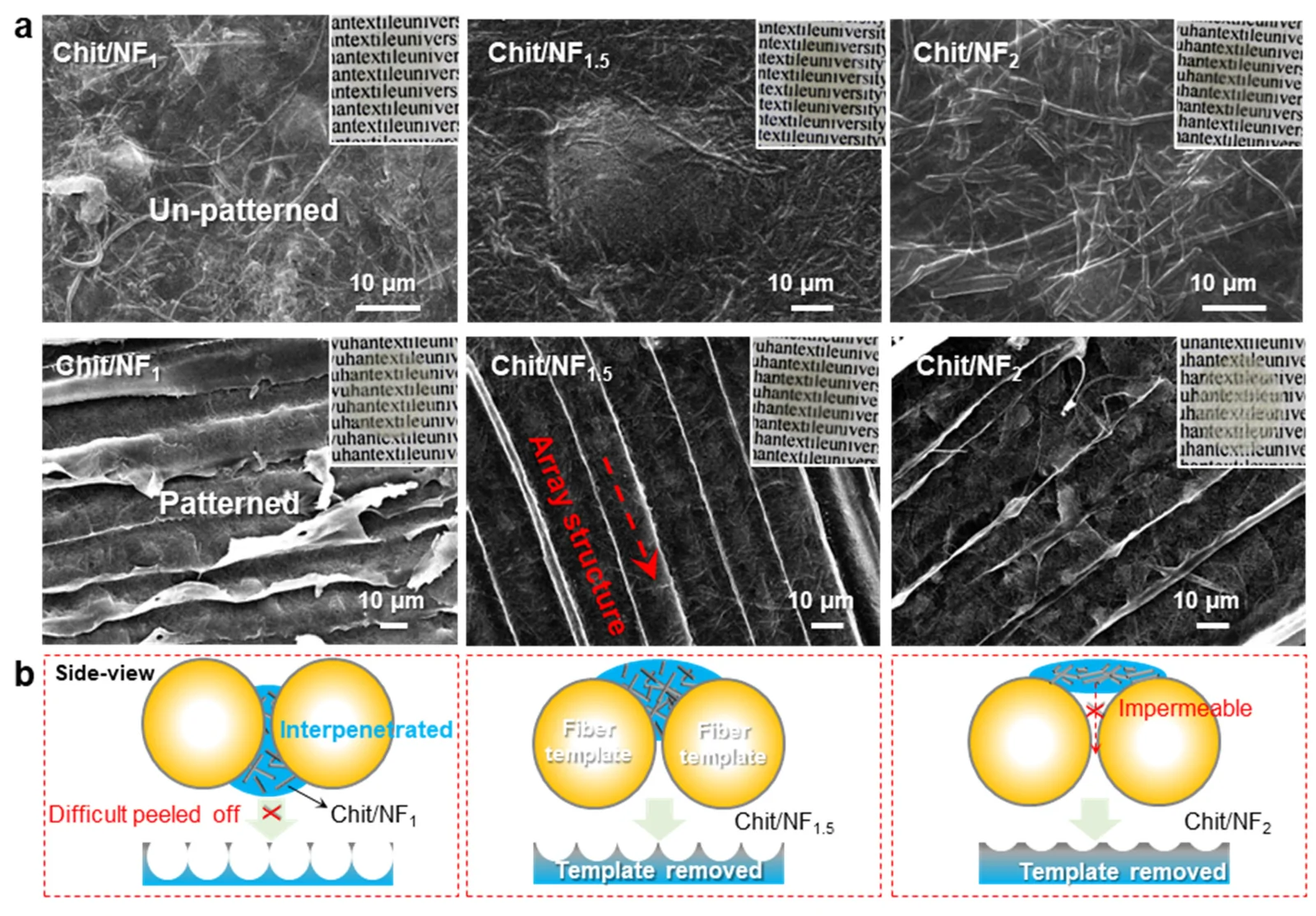
(**a**) SEM and optical images revealing the ordered array structures of Chit/NF nanocomposites prepared from varied Chit/NF ratios, with and without woven fabric templates. (**b**) Schematic illustration of the proposed formation mechanisms.

In this section, the influence of nanofiber concentrations and templates on the formulation of array structures is investigated. The SEM images in Figure 3a systematically compare un-patterned and patterned Chit/NF nanocomposites across three fiber concentrations. In the absence of a template, it can be observed that the composite film surface gradually exhibits a rough, fibrous stacking structure as the fiber content increases. As shown in the insets, the introduction of nanofibers progressively reduces the light transmittance of the composite films, suggesting that increasing fiber content promotes the formation of crosslinking points and thereby constructs a continuous three-dimensional network with higher fiber density. With the use of templates, well-defined micro/nanostructures appear on the film surface, accompanied by significant changes in optical transmittance. However, when the nanofiber concentration is either too low or too high, the aligned structures become irregular, which may be attributed to the fact that the nanofiber concentration greatly influences the viscosity of the mixed solution, thereby preventing the solution from properly integrating with the template fabric. The rheological results (Figure S2) show that the viscosity of the chitosan/NF mixed solutions gradually increases with increasing nanofiber concentration. The underlying mechanism is illustrated schematically in Figure 3b. For the Chit/NF_1_ with a relatively low fiber concentration, the low solution viscosity allows the mixed solution to penetrate through the fabric template and results in an interpenetrated network [43], which ultimately makes it impossible to peel off the composite film. Conversely, for the Chit/NF_2_ with a relatively high concentration, the high viscosity of the solution is unable to infiltrate the fiber interstitial spaces, resulting in incomplete structural replication. Therefore, the moderate NF concentration of Chit/NF_1.5_ enables a good preparation of the ordered surface array structure via the capillary-driven self-assembly within fabric templates. The sequential solvent-evaporation-induced co-solidification of fibers and matrix provides a low-cost alternative to complex alignment techniques (e.g., magnetic fields) for scalable nanofiber distribution control.

**Figure 4:**
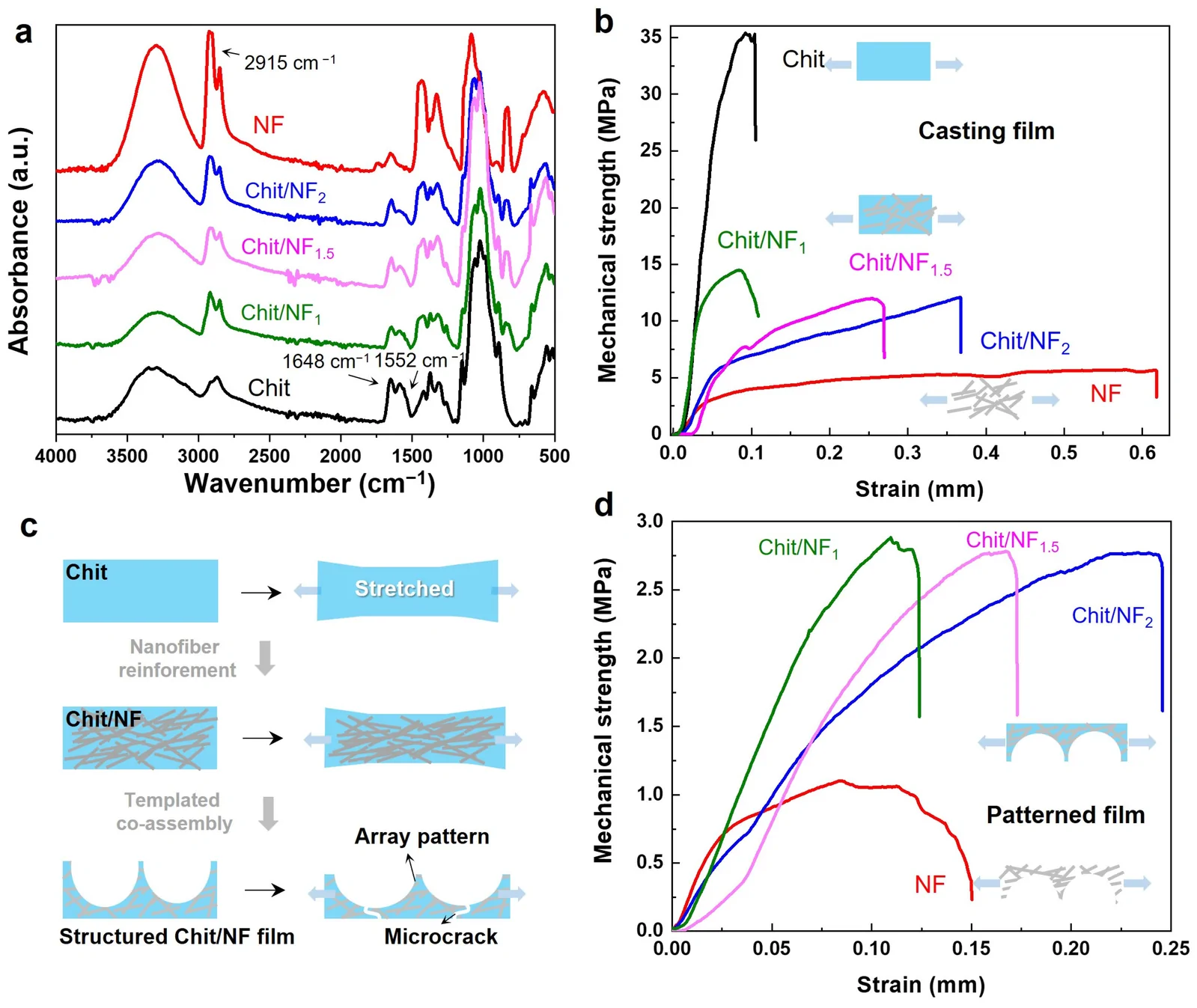
(**a**) FTIR spectra of the bare Chi and Chit/NF films prepared with different nanofiber concentrations. (**b**) Mechanical properties of Chit/NF films prepared by direct casting. (**c**) Schematic illustration of the effects of nanofiber reinforcement and templated co-deposition on the film mechanical properties. (**d**) Mechanical properties of Chit/NF films prepared by the fabric-templated co-deposition approach.

Figure 4a presents the FTIR spectra revealing the molecular interactions between the NF and Chit chains. The characteristic peak at 1324 cm^−1^ and 1450 cm^−1^ (bending vibration), 2850 cm^−1^ and 2928 cm^−1^ (stretching vibration) are assigned to the –C–H vibrations of NF [44]. The characteristic absorption bands of chitosan were observed at approximately 1648 cm^−1^ (Amide I) and 1552 cm^−1^ (Amide II), corresponding to the C=O stretching vibration and the N–H bending coupled with C–N stretching, respectively [45]. Detailed vibration bands and their assignments are summarized in Table S1. In the blended samples, a broad absorption band centered at ~3343 cm^−1^ (–O–H stretching vibration) indicates strong hydrogen-bonding interactions between chitosan (e.g., active amine groups) and the nanofibers (e.g., abundant hydroxyl groups), and this interaction becomes increasingly pronounced with increasing nanofiber content. All composite films exhibit the characteristic peaks of both components, suggesting that the blending process involves only physical interactions rather than chemical reactions. Figure 4b compares the mechanical properties of nanocomposite films with varying nanofiber contents fabricated via direct casting. The pristine chitosan film exhibits the highest tensile strength of up to 35 MPa, yet suffers from poor elongation at break, likely due to its high crystallinity and overly dense film structure. With increasing nanofiber incorporation, the tensile strength gradually decreases, as the nanofibers interfere with the self-crosslinking and crystallization of chitosan molecules. In contrast, the elongation at break progressively improves, which may be attributed to the large specific surface area of the nanofibers that enables reversible hydrogen bonding and intermolecular slippage, thereby enhancing the film flexibility.

The possible underlying mechanisms are shown in Figure 4c, demonstrating that nanofiber addition and template utilization can significantly modulate the mechanical performance of the composite films. While the introduction of nanofibers significantly reinforces the mechanical properties of the composite films through increased specific surface area and extensive hydrogen-bonding interactions and the fabric template effectively tailors the fiber distribution and surface array architectures, the relatively thinner regions at the base of the oriented grooves are prone to premature cracking under tensile loading. Such microcrack formation ultimately constrains the full realization of the anticipated mechanical enhancement. Figure 4d demonstrates that the fabric-templated nanofiber films exhibit a similar trend in mechanical property evolution. The pristine nanofiber film shows the lowest tensile strength due to weak interfacial interactions among the nanofibers. Chitosan serves as an effective binder, forming nanofiber-reinforced composite films with significantly improved mechanical properties. However, the enhancement in tensile strength is limited, as fracture tends to initiate preferentially at the thinnest regions of the films. Moreover, it should be noted that, in this work, the nanofibers are mainly used to adjust the solution viscosity to facilitate the formation of ordered surface structures. Owing to the weak interaction with the chitosan matrix in the absence of chemical crosslinking, they provide limited reinforcement but contribute to improved flexibility and deformation tolerance.

### 3.3. Constructing Conductive Array Structures for Flexible Wearable Materials

**Figure 5:**
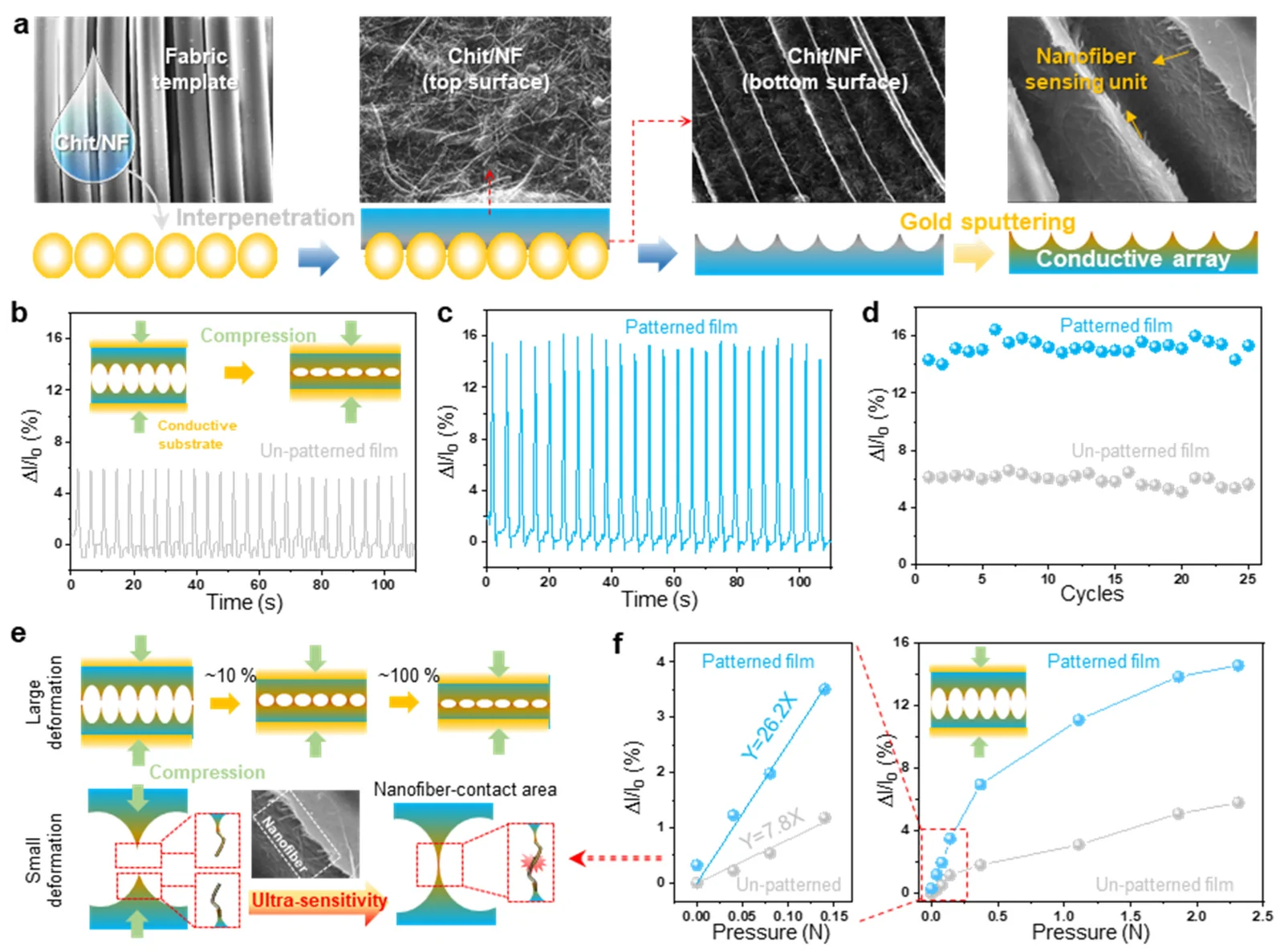
(**a**) Fabrication process for the conductive array-patterned nanocomposite films via Au sputtering. (**b**,**c**) Relative current variation (ΔI/I_0_) as a function of cyclic compression for films without (**b**) and with (**c**) surface array patterns, where ΔI denotes the current change and I_0_ is the initial current. (**d**) Peak current variation over 25 cycles demonstrating great reproducibility. (**e**) Schematic of the proposed sensing mechanism for array-patterned films under different deformation regimes. (**f**) Current response as a function of applied stress, revealing an ultra-high sensitivity in the low-stress region.

Finally, the composite films were rendered electrically conductive to assess their feasibility for wearable sensing applications. Figure 5a schematically illustrates the fabrication route for the conductive array films, along with corresponding SEM micrographs, where conductivity was imparted either by blending conductive nanofillers into the polymer matrix or via surface sputter-coating with a metallic gold coating layer. The brief Au sputtering process applied as a standard SEM conductive treatment with a coating thickness of several tens of nanometers would have only a minor effect on the mechanical properties, surface morphology, and flexibility of the patterned films. Next, we systematically evaluated the compressive cyclic piezoresistive performance to evaluate the effects of the surface array architecture. The periodic patterns inherited from the cotton fabric template serve as visual alignment guides, ensuring reliable face-to-face contact of the surface arrays. In the absence of the array structure (Figure 5b), the current variation remained as low as 6%, a marginal response attributable to the limited deformation of the flat film surface upon loading, which results in negligible contact-resistance modulation. In contrast, as shown in Figure 5c, the array-patterned counterpart delivered a substantially amplified current variation of 15.7% under identical conditions, translating into a 2.6-fold enhancement in sensing sensitivity. Moreover, this film-based array sensor exhibits rapid response and recovery times of 398 ms and 410 ms (Figure S3). Beyond instantaneous responsiveness, the long-term operational stability was interrogated through consecutive compression–release cycles. As compiled in Figure 5d, the peak currents over 25 cycles for both film configurations confirm robust stability, excellent reproducibility, and consistent signal recovery without detectable attenuation, revealing their durability for repeated use.

To confirm the origin of ultrasensitivity, Figure 5e proposes a novel nanofiber-contact-mediated strain-dependent sensing mechanism. Under large deformation, the ordered array elements undergo a pronounced increase in effective electrode contact area, generating substantial electrical signal variations that enable sensitive stress detection [46]. Under small deformation, however, a distinct regime comes into play rather than bulk compaction, and the nanofibers on channel walls are preferentially engaged, establishing localized conductive pathways that permit highly sensitive detection even in the low-stress regime. This dual-mechanism response, governed by the interplay between contact-area expansion and fiber-mediated percolation, underpins the overall superiority of the array architecture [47]. The quantitative relationship between current variation and applied stress is presented in Figure 5f. The cyclic compression tests confirm that the Au-sputtered composite films with ordered surface arrays maintain good structural stability and mechanical integrity under various compression strains (Figure S4). Within the force range of 0~0.15 N, the current signal rises steeply with increasing stress, affording a sensitivity approximately 3.4 times that of the un-patterned reference film. Collectively, these systematic characterizations not only substantiate the structural advantage conferred by the surface array design but also establish its practical potential for next-generation wearable sensing platforms and smart health-monitoring systems, where high sensitivity and mechanical durability are simultaneously demanded.

## 4. Conclusions

In summary, we have developed a fabric-templated co-deposition strategy for constructing chitosan/nanofiber composite films with ordered surface arrays. Woven cotton fabrics serve as soft templates, enabling facile replication of microscale groove architectures via capillary-driven infiltration and subsequent solvent evaporation. Optimal structural fidelity is achieved at a nanofiber content of 1.5 wt%, where strong hydrogen bonding stabilizes the chitosan matrix and tailors mechanical flexibility. Beyond structural control, this versatile platform allows for ready functionalization through straightforward co-deposition (e.g., FITC) or surface modification (e.g., conductive coatings), obviating complex procedures. After gold sputtering, the conductive array-patterned films exhibit superior piezoresistive performance, governed by a dual-mechanism response (e.g., contact-area enlargement under large deformation and nanofiber-mediated conduction under small deformation), which affords ultra-high sensitivity (3.4-fold enhancement in the low-stress regime), excellent cyclic stability, and reliable reproducibility. This work establishes a low-cost, scalable route for constructing ordered surface microstructures without relying on lithographic techniques, holding promise for applications in wearable strain sensors, health monitoring, and transdermal drug delivery.

## Data Availability

The original contributions presented in this study are included in the article; further inquiries can be directed to the corresponding authors.

## Supplementary Materials

The following supporting information can be downloaded at: https://www.mdpi.com/article/10.3390/polym18182278/s1.
