# Supplementary material for: Fabric-Templated Co-Deposition of Nanofiber-Reinforced Composite Films with Ordered Surface Arrays for Ultrasensitive Wearable Sensing
Source: Polymers (Basel). 2026 Sep 17;18(18):2278. doi: 10.3390/polym18182278 (PMC13611419; doi:10.3390/polym18182278)
Supplement: Supplementary file 1 [file polymers-18-02278-s001.zip › polymers-4484480-supplementary.pdf]

Supporting Information

for

Fabric-Templated Co-Deposition of Nanofiber-Reinforced Composite Films with Ordered Surface Arrays for Ultrasensitive Wearable Sensing

Yixiao Wu[1], Yuchen Luo[1], Ziyi Xiong[1], Sile He[2], Lin Yang[1], Chong Zhao[1] and Kun Yan[3]**

[1] School of Chemistry and Environmental Engineering, Wuhan Polytechnic University, Wuhan, Hubei 430023, China

[2] School of Environmental and Chemical Engineering, Zhaoqing University, Zhaoqing, Guangdong 526061, China

[3] Key Laboratory of Textile Fiber & Product, Ministry of Education, Wuhan Textile University, Wuhan 430200, China

* Correspondence: hesile@zqu.edu.cn (S.H.); kyan@wtu.edu.cn (K.Y.)

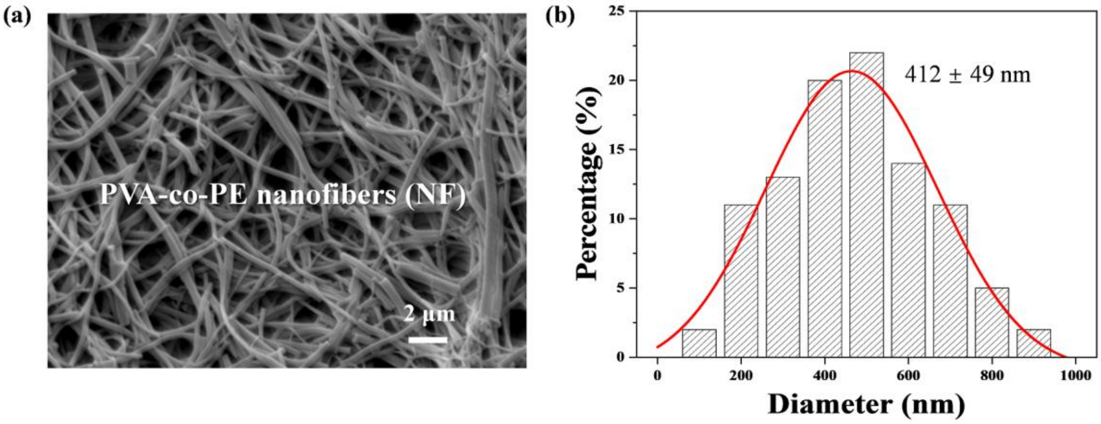

Figure. S1 (a) Scanning electron microscopy (SEM) image of the PVA-co-PE nanofibers, and (b) the corresponding diameter distribution histogram.

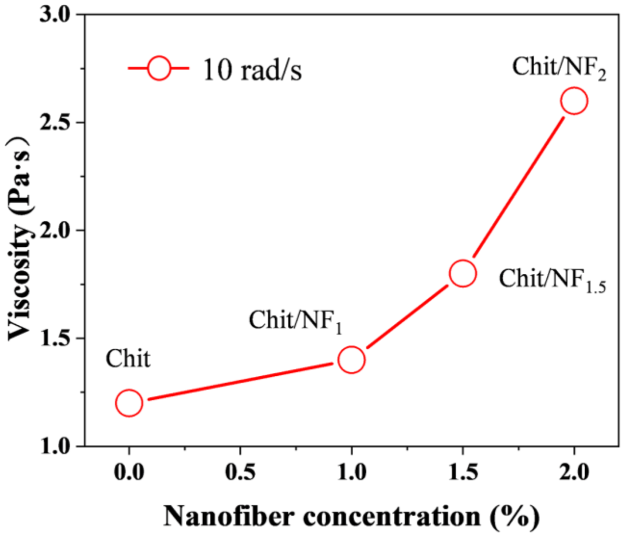

Figure. S2 Rheological characterization revealing the effect of nanofiber concentration on the viscosity of the chitosan solution.

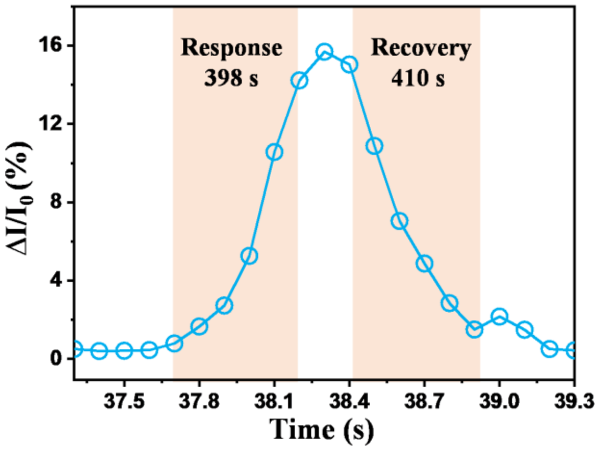

Figure. S3 Quantitative analysis of the film-based array sensor, exhibiting response and recovery times of 398 ms and 410 ms, respectively.

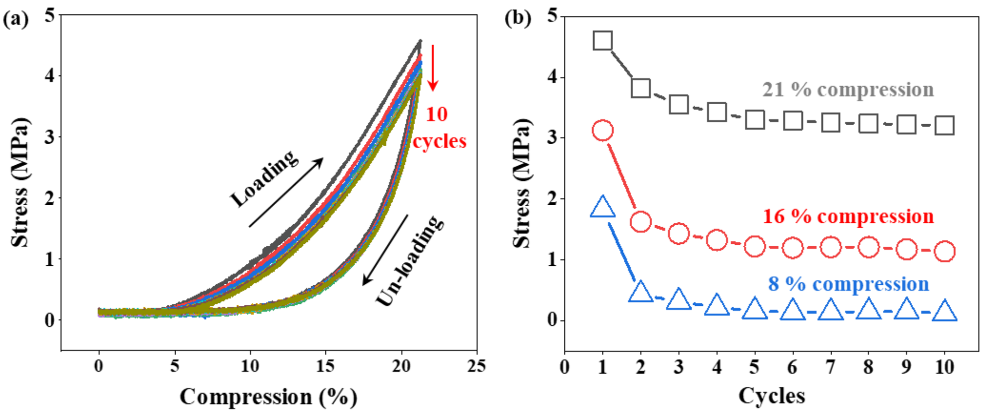

Figure. S4 (a) Cyclic compression mechanical curves of the nanocomposite film featuring ordered surface array structures over 10 loading–unloading cycles. (b) Statistical comparison of mechanical stability under different compression strains.

Table S1. Summary of characteristic FTIR vibration bands and their assignments.

**Wavenumber**	**Assignment**	**Vibration mode**	**Reference**
~3440 cm^{-1}	O–H / N–H	Stretching	[39]
~2928 / 2850 cm^{-1}	C–H	Asymmetric / symmetric stretching	[39]
~1648 (Amide I) cm^{-1}	C=O	Stretching	[40]
~1552 cm^{-1} (Amide II)	N–H bending / C–N	Bending / stretching	[40]
~1450 / 1324 cm^{-1}	C–H	Bending	[39]
